# Subtotal Pancreatectomy for Cancer: Closure of the Pancreatic Remnant With Staplers

**DOI:** 10.1155/1990/73475

**Published:** 1990

**Authors:** Bo Ahrén, Karl-G. Tranberg, Åke Andrén-Sandberg, Stig Bengmark

**Affiliations:** 1 Department of Surgery Lund University Lund Sweden; 2 Department of Surgery Lund University Lund S-221 85 Sweden

## Abstract

This paper presents a 2-year series of 26 consecutive pancreatectomies for periampullary cancer where the pancreatic tail was closed with a stapler in order to avoid complications related to a pancreatico-digestive anastomosis. The follow-up period was 14 months or more. Seven patients developed operative complications. Pancreatic fistulas developed in 3 patients. The fistulas closed spontaneously in 2 of the patients after 2-4 months, lntraabdominal abscesses developed in 4 patients and required surgical drainage. In 1 of these patients, the abscess eroded a large vessel with a fatal outcome resulting in an operative mortality rate of 3.8%. A transient postoperative gastric stasis was observed in seven patients. Postoperative hospital median stay was 27 days (range 10–83 days). Eighteeen patients have died after 4–30 months in recurrent disease and seven patients are alive after a follow-up period of 15–29 months. Pancreatic endocrine function seemed well preserved; diabetes mellitus has developed in only one patient. In conclusion, it appears that subtotal pancreatectomy with closure of the pancreatic remnant with staples gives a low morbidity and mortality. Although the conclusion should be tempered by the small number of patients, the results justify continued evaluation of this technique with long-term follow-up.


*HPB Surgery*, 1990, Vol. 2, pp. 29–39
Reprints available directly from the publisher
Photocopying permitted by license only

© 1990 Harwood Academic Publishers GmbH
Printed in the United Kingdom

### SUBTOTAL PANCREATECTOMY FOR CANCER: CLOSURE OF THE PANCREATIC REMNANT WITH STAPLERS

BO AHRÉN, KARL-G. TRANBERG, ÅKE ANDRÉN-SANDBERG, and STIG BENGMARK

*Department of Surgery, Lund University, Lund, Sweden*

*(Received 25 August 1988)*

This paper presents a 2-year series of 26 consecutive pancreatectomies for periampullary cancer where the pancreatic tail was closed with a stapler in order to avoid complications related to a pancreatico-digestive anastomosis. The follow-up period was 14 months or more. Seven patients developed operative complications. Pancreatic fistulas developed in 3 patients. The fistulas closed spontaneously in 2 of the patients after 2–4 months. Intraabdominal abscesses developed in 4 patients and required surgical drainage. In 1 of these patients, the abscess eroded a large vessel with a fatal outcome resulting in an operative mortality rate of 3.8%. A transient postoperative gastric stasis was observed in seven patients. Postoperative hospital median stay was 27 days (range 10–83 days). Eighteeen patients have died after 4–30 months in recurrent disease and seven patients are alive after a follow-up period of 15–29 months. Pancreatic endocrine function seemed well preserved; diabetes mellitus has developed in only one patient. In conclusion, it appears that subtotal pancreatectomy with closure of the pancreatic remnant with staples gives a low morbidity and mortality. Although the conclusion should be tempered by the small number of patients, the results justify continued evaluation of this technique with long-term follow-up.

Despite aggressive surgical attempts, the prognosis of pancreatic carcinoma has remained poor [1–7]. Already at the time of diagnosis, most patients have distant metastases or are otherwise not fit for major surgery [5,8]. For those whose tumour is considered resectable, the operative approach has been a matter of discussion, and both subtotal and total pancreatectomy have been advocated [5,8–16].

Insufficiency of a pancreato-digestive anastomosis is a common and frequently lethal complication after pancreatic surgery, because of leakage of pancreatic secretion with abscess and/or fistula formation[6,17–20]. Therefore, attempts to improve the results of partial pancreatectomy must be directed towards this weakness of the operative procedure. Several technical solutions have been presented: mucosa to mucosa-anastomosis; splints in the anastomosis; obstruction of the outflow of pancreatic juice by means of ligation; stapling of the pancreatic remnant, instillation of prolamine (Ethibloc[R]) into the pancreatic duct; open drainage; or double jejunal loop [21–26]. This report presents our preliminary experience of an extended pancreatic resection combined with stapling the pancreatic remnant without reestablishing pancreato- digestive continuity.

Correspondence to: Bo Ahrén, Dr. Department of Surgery, Lund University S-221 85 LUND, Sweden.

### PATIENTS

During a 2 year period, July 1985–June 1987, a total of 69 patients with newly diagnosed carcinoma of the exocrine pancreatic head (n = 56), the ampulla of Vater (n = 8), or the distal choledochal duct (n = 5) were treated at the Department of Surgery in Lund. Of these, 26 patients (38%) were considered to have a resectable tumour and underwent subtotal pancreatectomy with duodenectomy. Seventeen patients had carcinoma of the pancreatic head, 7 had cancer of the Vaterian papilla, and 2 had a choledochal carcinoma. The men age of the resected patients was 6310 (SD) years (range 40–80 years). The operation was considered radical if as judged after the operation all malignant tissue had been removed. Otherwise, the operation was considered to be palliative.

### OPERATIVE PROCEDURE

The operation starts with extensive dissection of the retroduodenal region, the inferior vena cava from above the left renal vein and 10 cm downward, and of the hepatoduodenal and gastrohepatic ligaments. The gallbladder is excised. All tissue but the major arteries, the portal vein and the bile ducts, are removed from the hilum of the liver down to the origin of the coeliac artery. All identifiable lymph glands are sent for separate histologic examination. The coeliac plexus is included in the resection. The pancreas is divided with stapler above the portal vein. The pancreas to the right of the portal vein is removed together with the duodenum, adjacent retroperitoneal tissue, two thirds of the stomach, the proximal jejunum and the extrahepatic biliary tree approximately one cm below the junction of the hepatic ducts. Care is taken to remove all tissue on the posterior and right sides of the superior mesenteric and portal veins and the superior mesenteric artery, from the hilum of the liver and the aorta, respectively, down to the first jejunal branches. About half of the remaining pancreas (approximately the body) is dissected from the splenic vessels before the pancreas is again stapled off, with a TA55 instrument (the first 14 patients in the series) or RL60 (the last 12 patients in the series), 6–7 cm from the end of the tail. In addition, the pancreatic duct is suture-ligated and, in some cases, a running 2-0 Prolene suture is used to reinforce the closure of the severed end of the pancreas. Hepatojejunostomy is fashioned with a 60 cm jejunal Roux-en-Y-loop end to side and followed gastrojejunostomy to the posterior or anterior side of the stomach; the end of the Roux loop is placed subcutaneously. A separate incision was made for placement of a rubber drain down to the peripancreatic area.

### FOLLOW UP

The patients have been regularly followed by the operating surgeon. The last patient in this series has been followed now for 14 months. The regular follow-up has included computed tomography of the upper abdomen every third month.

### RESULTS

Fifteen of the 26 patients had an over-all uneventful postoperative course. Median postoperative hospital stay was 27 days (range 10–83 days; Table 1). In three patients, the drainage from the incision used for the abdominal drain continued more than 10 days without the existence of any abscess; these patients were defined as having developed pancreatocutaneous fistulas. This complication did not, however, cause significant morbidity. The fistulas closed spontaneously after 2–4 months. Secretion from the fistulas was collected in a colostomy bag and did not cause irritation of the skin or other discomfort. Frequent postoperative estimations did not disclose elevation of serum amylase levels. Four patients developed intraabdominal abscesses which required surgical drainage. These abscesses were located peripancreatically and were found to contain infected material. In one of these patients, the abscess eroded a large vessel which resulted in a profound hemorrhage with a fatal outcome. This was the only mortality in this series of patients, yielding a mortality rate of 3.8%.

Most patients showed some signs of delayed gastric and/or intestinal emptying which was the main reason behind the relatively long hospital stay. In seven of them, gastric or jejunal dysfunction required treatment with nasogastric tube and intravenous infusion more than 10 days because of inability to ingest more than minimal amounts of fluid. No pathological findings could be demonstrated in these patients at contrast meal studies or gastroscopy. The condition improved spontaneously in six patients who were discharged after 36–83 postoperative days, respectively. The seventh patient (No 3 in Table 1) was reoperated on at the 36th postoperative day. The gastro-jejunal anastomosis was found to be intact but a hard inflammatory mass around the remnant pancreas was found to involve a few centimeters of the posterior side of the jejunum close to the gastro-entero anastomosis. Following resection of the short segment of the affected jejunum, the patient had an uneventful course.

Only one patient developed diabetes (No 2 in Table 1); it occurred 8 months after the operation and required regular insulin injections. Four patients had been on antidiabetic treatment before the operation, one with insulin, the other three with oral hypoglycaemic agents. The diabetes has postoperatively been stable in these four patients.

Seven patients are alive (Table 1). Median follow-up period for these patients is 18 months (range 15–29 months). Eighteen patients have died at 4–30 months after the operation due to generalized disease.

### DISCUSSION

Arguments for total pancreatectomy in the treatment of pancreatic cancer include the fact that the disease is potentially multifocal, that a more radical resection of the regional lymph nodes may be done, and that the degree of complications might be lower than after partial pancreatectomy, mainly because a pancreato-digestive anastomosis is avoided [5,8–16]. Proponents of total pancreatectomy also argue that the resulting diabetes is no longer a great problem. Besides, many patients have diabetes already at the time of diagnosis and will not live long enough to develop secondary complications to the diabetes.

Others authors, however, consider partial pancreatectomy to be the treatment of choice[5,13–15]. This attitude is corroborated by recent reports indicating that partial and total pancreatectomy are similar with respect to operative mortality and morbidity as well as long-term survival[6,13,15,16]. Partial pancreatectomy with a pancreato- digestive anastomosis also preserves the spleen and the external pancreatic secretion and does not usually produce diabetes.

**Table 1** Clinical Material

| *Patient* | *Op. Month* | *Age (yrs)* | *Sex* | *Origin of Cancer* | *Maximal Diameter of tumour* (cm) | *Lymph node involvement* | *Capsular involvement* | *Type of operation* (classified postoperatively) | *Complication* | *Postoperative hospital stay (days)* | *Follow-up (months)* |
|---|---|---|---|---|---|---|---|---|---|---|---|
| 1 | 8507 | 53 | M | Pancreas | 1.0 | No | No | Radical | | 27 | 22 (dead) |
| 2 | 8509 | 67 | F | Pancreas | 1.5 | Yes | Yes | Radical | | 22 | 30 (dead) |
| 3 | 8601 | 68 | F | Pancreas | 2.0 | No | No | Radical | Fistula + Gatric Stasis | 79 | 14 (dead) |
| 4 | 8603 | 72 | M | Pancreas | 2.0 | No | No | Radical | | 28 | 29 |
| 5 | 8603 | 77 | F | Pancreas | 4.0 | Yes | Yes | Radical | Fistula + Gastric Stasis | 35 | 22 (dead) |
| 6 | 8604 | 40 | M | Pancreas | 6.0 | No | No | Radical | Abscess + Fatal hemmorhage | 40 | 1 (dead) |
| 7 | 8607 | 46 | M | Pancreas | 4.0 | Yes | Yes | Palliative | Gastric Stasis | 83 | 4 (dead) |
| 8 | 8608 | 53 | M | Pancreas | 2.0 | Yes | Yes | Palliative | | 15 | 8 (dead) |
| 9 | 8612 | 49 | M | Pancreas | 3.5 | No | No | Radical | | 15 | 21 |
| 10 | 8612 | 55 | M | Pancreas | 3.0 | Yes | Yes | Radical | | 34 | 21 |
| 11 | 8702 | 71 | M | Pancreas | 3.5 | No | No | Radical | | 15 | 18 |
| 12 | 8702 | 47 | F | Pancreas | 3.0 | Yes | Yes | Palliative | | 25 | 4 (dead) |
| 13 | 8703 | 60 | M | Pancreas | 3.5 | No | No | Radical | Abscess | 32 | 16 (dead) |
| 14 | 8703 | 64 | M | Pancreas | 4.0 | Yes | Yes | Palliative | Gastric Stasis | 36 | 17 |
| 15 | 8703 | 56 | M | Pancreas | 5.0 | Yes | Yes | Palliative | Abscess + Gastric Stasis | 61 | 11 (dead) |
| 16 | 8703 | 60 | F | Pancreas | 6.0 | No | No | Radical | | 15 | 5 (dead) |
| 17 | 8705 | 72 | M | Pancreas | 3.5 | No | No | Radical | | 10 | 15 |
| 18 | 8512 | 72 | F | Pap Vateri | 4.0 | Yes | No | Palliative | Gastric Stasis | 47 | 10 (dead) |
| 19 | 8512 | 64 | F | Pap Vateri | 2.5 | Yes | Yes | Palliative | | 24 | 16 (dead) |
| 20 | 8601 | 67 | M | Pap Vateri | 2.0 | Yes | Yes | Radical | | 24 | 17 (dead) |
| 21 | 8601 | 68 | F | Pap Vateri | 7.0 | No | No | Radical | | 29 | 7 (dead) |
| 22 | 8604 | 77 | M | Pap Vateri | 1.5 | Yes | No | Radical | Gastric Stasis | 57 | 25 (dead) |
| 23 | 8605 | 80 | M | Pap Vateri | 6.0 | Yes | Yes | Palliative | Fistula | 16 | 6 (dead) |
| 24 | 8703 | 60 | M | Pap Vateri | 3.5 | No | No | Radical | Abscess + Gastric Stasis | 42 | 17 |
| 25 | 8608 | 68 | F | Choledochus | 2.2 | Yes | Yes | Palliative | | 15 | 14 (dead) |
| 26 | 8702 | 61 | M | Choledochus | 1.5 | No | No | Radical | | 21 | 10 (dead) |

In recent reports, pancreato-duodenectomy for pancreatic cancer has had a 2.5–10% hospital mortality rate and an overall postoperative morbidity of 20–30%[4–7,17,27–31]. Most complications have been related to the pancreato-digestive anastomosis[6,7,17–20].

The technique used by us is not encumbered with the complications of a pancreato-digestive anastomosis. Instead, we would expect an increased frequency of pancreatic fistulas[6,17–20], likely, though, to be relatively non-aggressive since most pancreatic enzymes are not activated. However, of our 26 patients operated during a 2 year period, only three patients developed pancreatic fistula (12%), and these fistulas did not cause significant morbidity. This frequency of pancreatic fistulation is lower than in earlier reports of subtotal pancreatectomy with closure of the pancreatic remnant [17,32]. One possible explanation for this difference is that closure of the pancreatic remnant has previously been performed only when it was considered too hazardous to perform a pancreato- jejunostomy. Another explanation for the comparatively low frequency of fistulas in our series might be the small pancreatic remnant. However, besides the three patients developing pancreatic fistula, another 4 developed peripancreatic abscesses, giving a total complication rate of 27% in the immediate postoperative period.

One can speculate that it might be possible to further reduce the frequency of pancreatic fistulas by the use of somatostatin or somatostatin analogues in the immediate postoperative period [33,34], or by the use of more suitable stapling devices.

Seven of our patients developed a transient period of delayed gastric emptying and a delayed recovery of gastrointestinal function. This finding confirms earlier reports on subtotal pancreatectomy with pancreatodigestive anastomosis [35,36]. The postoperative gastric stasis might be explained by the slower mobility of jejunum as compared to duodenum, to operative damage of the blood or nerve supply to the gastric remnant or to the jejunum, to peripancreatic inflammatory changes, or to hormonal changes after the operation [35]. This issue is unsettled and it would therefore be of interest to study the physiological consequences of this type of operation.

In the present series, staplers originally designed for stapling the gastrointestinal tract, like TA55, were used. These staple the important ducts but usually crush the pancreatic capsule. In our last eleven operations, a stapler instrument with a larger distance within the staples, RL60, have been used. Hereby the pancreas may be safely stapled with an intact pancreatic capsule. However, it is obvious that different stapler-devices are needed due to the differences in size and degree of fibrosis of the pancreatic tail. In the future, staplers especially designed for solid organ stapling may be available.

One patient developed overt diabetes 8 months after the operation. This compares well with the reported 12.5% incidence of diabetes after partial pancreato-duodenectomy with pancreato-digestive anastomosis [11]. Whether other patients developed impaired glucose tolerance can not be established since glucose tolerance test was not performed routinely after the operation. The reason why diabetes does not usually evolve after partial pancreatectomy is probably explained by studies indicating that more than 70% of the pancreas can be removed from an otherwise healthy gland without the development of diabetes [37,38]. All patients operated on by this technique develop pancreatic insufficiency and are given pancreatic enzymes. After the postoperative phase, bowel function has been normal. In addition, the weight of the patients has been stable, and some but not all patients have regained their pre-illness weight.

In summary, subtotal pancreatectomy with closure of the pancreatic remnant has the advantage of leaving healthy endocrine pancreatic tissue and the spleen and avoids a potentially hazardous pancreato-digestive anastomosis. The low operative mortality and the good quality of life after the operation encourage us to continue the use of this technique. It is too early for an accurate evaluation of the long-term results, but the long- term survival does not seem to be prolonged, at least not when compared to historical series [12].

### *References*

1. Longmire, Jr W.P. The vicissitudes of pancreatic surgery. *Am. J. Surg.* **147**: 17–24, 1984.
2. Moossa, A.R. Surgical treatment of pancreatic cancer. *Am. J. Surg.* **152**: 503–504, 1984.
3. Matsumo, S., Sato, T. Surgical treatment for carcinoma of the pancreas. Experience in 272 patients. *Am J Surg* **152**: 499– 503, 1986.
4. Kairaluoma, M.I., Kiviniemi, H., Ståhlberg, M. Pancreatic resection for carcinoma of the pancreas and the periampullary region in patients over 70 years of age. *Brit. J. Surg.* **74**: 116–118, 1987.
5. Trede, M. Treatment of pancreatic carcinoma: the surgeon's dilemma. *Brit. J. Surg.* **74**: 79–80, 1987.
6. Tarazi, R.Y., Hermann, R.E., Vogt, D.P., Hoerr, S.O., Esselstyn, Jr C.B., Cooperman, A.M., Steiger, E., Grundfest, S. Results of surgical treatment of periampullary tumors: A thirty-five-year experience. *Surgery* **100**: 716–721, 1986.
7. Sirinek, K.R., Aust, J.B. Pancreatic cancer: Continuing diagnostic and therapeutic dilemma. *Surg. Clin. N. Amer.* **66**: 757–777, 1986.
8. Moossa, A.R. Pancreatic cancer: approach to diagnosis, selection for surgery and choice of operation. *Cancer* **50**: 2689–2698, 1982.
9. Trede, M. The surgical treatment of pancreatic carcinoma. *Surgery* **97**: 28–35, 1985.
10. Collins, Jr. J.J, Craighead, J.E., Brooks, J.R. Rationale for total pancreatectomy for carcinoma of the pancreatic head. *N. Engl. J. Med.* **274**: 599–602, 1966.
11. Ihse, I., Lilja, P., Arnesjö, B., Bengmark, S. Total pancreatectomy for cancer – an appraisal of 65 cases. *Ann. Surg.* **186**: 675–680, 1977.
12. Andrén-Sandberg, Å, Ihse, I. Factors influencing survival after total pancreatectomy in patients with pancreatic cancer. *Ann. Surg.* **198**: 605–610, 1983.
13. Edis, A.J., Kiernam, P.D., Taylor, A.F. Attempted curative resection of ductal carcinoma of the pancreas: a review of the Mayo Clinic experience. *Mayo Clinic Proc.* **55**: 531–534, 1980.
14. Heerden, J.A. Pancreatic resection for carcinoma of the pancreas: Whipple versus total pancreatectomy. An institutional perspective. *World. J. Surg.* **8**: 880–888, 1984.
15. Crace, P.A., Pitt, H.A., Tompkins, R.K., *et al.* Decreased morbidity and mortality after pancreato-duodenectomy. *Am. J. Surgery* **151**: 141–149, 1986.
16. Longmire, W.P. Carcinoma of the pancreas: palliative operation, Whipple procedure or total pancreatectomy. *World J. Surg.* **8**: 872–879, 1984.
17. Papachristou, D.N., Fortner, J.G. Pancreatic fistula complicating pancreatectomy for malignant disease. *Brit. J. Surg.* **68**: 238–240, 1981.
18. Papachristou, D.N., Fortner, J.G. Management of the pancreatic remnant in pancreatoduodenectomy. *J. Surg. Oncol.* **18**: 1–7, 1981.
19. Balasegaram, M. Carcinoma of the periampullary region. A review of a personal series of 87 patients *Brit. J. Surg.* **63**: 532–537, 1976.
20. Langer, B., Lipson, R., McHatie, J.D. Periampullary tumours: Advances in diagnosis and surgical treatment. *Can. J. Surg.* **22**: 34–37, 1979.
21. Goldsmith, H.S., Ghosh, B.C., Huvos, A.G. Ligation versus implantation of the pancreatic duct after pancreatectomy. *Surg. Gynecol. Obstet.* **132**: 87–92, 1971.
22. Kummerle, F., Rickert, K. Surgical treatment of pancreatic cancer. *World. J. Surgery.* **8**: 889–894, 1984.
23. Kerremans, R.P., Penninckx, F.M., Fevery, J., DeGroote, J. Subtotal resection of the head of the pancreas combined with ductal obliteration of the distal pancreas in chronic pancreatitis. *Ann. Surg.* **205**: 240–245, 1987.
24. Lygidakis, N.J., Brummelkamp, W.H. A new approach for the reconstruction of continuity of the alimentary tract after pancreaticoduodenectomy. *Surg. Gynecol. Obstet.* **160**: 453–458, 1985.
25. Fritsch, A Wiederherstellungsoperation nach Duodenopankreatektomie. *Langenbecks Arch. Chir.* **366**: 249– 255, 1985.
26. Sutherland, D.E.R., Baumgartner, D., Najarian, J.S. Free intraperitoneal drainage of segmental pancreas grafts: Clinical and experimental observations on technical aspects. *Transpl. Proc.* **12**: 26–31, 1980.
27. Kairaluoma, M.I., Partio, E., Ståhlberg, M., Laitinen, S. Pancreatic and periampullary carcinoma. *Ann. Chir. Gynaecol.* **73**: 199–205, 1984.

28. Lee, Y.T. Surgery for carcinoma of the pancreas and periampullary structures: Complications of resectional and palliative procedures. *J. Surg. Oncol.* **27**: 280–285, 1984.
29. Grace, P.A., Pitt, H.A., Tompkins, R.K., *et al.* Decreased morbidity and mortality after pancreatoduodenectomy. *Am. J. Surg.* **151**: 141–149, 1986.
30. Warren, K.W., Choe, D.S., Plaza, J., Relihan, M. Results of radical resection for periampullary cancer. *Ann. Surg.* **181**: 534–540, 1975.
31. Braasch, J.W., Gray, B.N. Considerations that lower pancreatoduodenectomy mortality. *Am. J. Surg.* **133**: 480–484, 1977.
32. Dencker, H. The management of the pancreatic duct at pancreatectomy. *Acta. Chir. Scand.* **138**: 620–623, 1972.
33. Mulvihill, S., Pappas, T.N., Passaro, E., Debas, H.T. The use of somatostatin and its analogs in the treatment of surgical disorders. *Surgery* **98**: 467–476, 1986.
34. Ahrén, B., Tranberg, K.G., Bengmark, S. Treatment of pancreatic fistula with the somatostatin analogue SMS 201-995. *Brit. J. Surg.* **65**: 718, 1988.
35. Warshaw, A.L., Torchiana, D.L. Delayed gastric emptying after pylorus preserving pancreatoduodenectomy. *Surg. Gynecol. Obstet.* **160**: 1–4, 1985.
36. Itani, K.M.F., Coleman, R.E., Akwari, O.E., Meyers, W.C. Pylorus-preserving pancreatoduodenectomy. A clinical and physiologic appraisal. *Ann. Surg.* **204**: 655–664, 1986.
37. Bonner-Weir, S., Trent, D.F., Weir, G.C. Partial pancreatectomy in the rat and subsequent defect in glucose-induced insulin release. *J. Clin. Invest. 1983*; **71**: 1544–1553, 1983.
38. Mizumoto, R., Yano, T., Sekoguchi, T., Kawarada, Y. Resectability of the pancreas without producing diabetes, with special reference to pancreatic regeneration. *Int. J. Pancreatol.* **1**: 185–194, 1986.

(Accepted by S. Bengmark 26 April 1989)

### INVITED COMMENTARY

Attempting to prevent the troublesome complication of leakage of the pancreaticojejunostomy, the authors have sacrificed exocrine pancreatic function by stapling closed the ductal system of the pancreatic remnant. In this they have not been entirely successful as the resulting incidence of temporary pancreatic fistula has been 12%.

Using the expression of Dr. Kenneth Warren, the commentators prefer a "precise anastomosis" of the pancreatic duct, per se, to a tiny opening in the side of the jejunal Roux Y. Utilizing 5.0 prolene sutures for the anastomosis and utilizing, temporarily, a small percutaneous catheter through the anastomosis, external pancreatic fistulae have essentially disappeared.

The authors have not recommended pancreatic stapling in patients undergoing resection for benign pancreatic disease in whom the life expectancy would be longer. In such patients, the metabolic deficits, including the possible delayed loss of islet cell function, might prove too high a price.

In eight patients (31%), gastric stasis after operation proved to be a significant complication. The commentators have had a similar experience. Doberneck[1] described gastric stasis as a major complication in 15 of 57 patients (26%) undergoing palliative gastrojejunostomy and cholecystojejunostomy for unresectable pancreatic cancer. They reviewed the relatively limited, earlier literature on the subject and attributed the problem to technical factors.

The commentators have also found gastric stasis a greater problem in the patients with bypass gastrojejunostomies (unresected pancreatic cancer) than with gastrojejunostomy after the Whipple resection, although the latter group is not free of the problem.

If the biliary jejunal-anastomosis is made upstream from the gastrojejunal anastomosis, it is difficult to position the efferent limb of the gastrojejunonostomy along the lessor curvature of the stomach — its "magenstrasse". Positioning the efferent limb on the greater curvature of the stomach may provide a transient impediment to gastric emptying. (We do not utilize vagotomy after pancreatic resection or bypass.) Repeatedly, endoscopy or reoperation has shown no anastomatic obstruction. The passage of time has inevitably permitted resumption of adequate anastomotic function. Meanwhile, the commentators often place a feeding jejunostomy and sometimes a decompressive gastrostomy tube as an adjunct to palliative bypasses.

Ranitidine (Zantac), widely used postoperatively as a hydrogen ion blocker, has been observed experimentally to retard gastric emptying in man[2]. Reglan, utilized to promote gastric motility, has not solved the problem.

Meanwhile, all of our related patients have baseline radionuclear scans of gastric emptying preoperatively and serially postoperatively, utilizing scrambled eggs orally into which is mixed a technicum - 99 sulphur colloid. Transient, but troublesome postoperative gastric stasis is frequent. Although gastric atony is obviously a factor, no study has yet proved whether the fundamental problem has a technical or physiological basis.

*References*

1. Doberneck, R.C. and Berndt, G.A., Delayed gastric emptying after palliative gastrojejunostomy for carcinoma of the pancreas. *Arch Surg.* Vol. **122:** 827–829, 1987.
2. Scarpignato, C. and Bertaccini, G., Different effects of cimetidine and ranitidine on gastric emptying in rats and man. *Agents and Actions,* Vol. **12:1/2,** 172–173, 1982.

John M. Howard
Professor of Surgery
and
Hollis W. Merrick
Associate Professor of Surgery
Medical College of Ohio USA

### INVITED COMMENTARY

The paper by Ahren and others[1] shows once again that pancreatoduodenectomy for cancer can be performed with low mortality. In the last decade hospital mortality after this procedure has decreased below 5%[2], and mortality of more than 10% is regarded as unacceptable. One should be aware that most reports, such as the one by Ahren et al[1] come from well-known centres, with special interest and experience in pancreatic surgery, and although excellent results can be achieved by the occasional "pancreatectomist"[3], it is fair to say that the results of most unpublished series are not as good.

Hospital mortality is generally considered to be the yardstick of surgical quality, but morbidity and long-term sequellae may be just as important since they affect to a large extent the quality of life after pancreatoduodenectomy. Serious postoperative complications are frequent and occur in about 30%, leading to reoperations, long hospitalisation and high costs[4]. Breakdown of the pancreato-enteric anastomosis is the most feared complication that occurs in about 12%[3]. The mortality of this complication can be as high as 40%[5] but adequate conservative treatment[2]

or aggressive surgery, completion performing a so-called completion pancreatectomy[4], dramatically decreased mortality. Various surgical techniques have been used to connect the pancreatic remnant to the gastrointestinal tract. The pancreatojejunostomy is probably the most physiological and the most frequently used technique. Either a mucosa to mucosa anastomosis or an invaginating or "dunking" anastomosis is preferred. Long internal — external splints or short splints are used or splints are abandoned altogether since they are thought to lead to obstruction and to have an adverse effect. Duration of splinting has also been discussed. Separating the bile duct and the pancreatic duct by separate jejunal loops as advocated by Macado *et al.*[7] does not lower the chance of leakage but less serious complications result since the pancreatic juice is not activated[8]. Pancreatogastrostomy seems an interesting alternative, having very high success rate. In one collective series where pancreatogastrostomy was performed only one anastomotic breakdown in 134 patients was seen[9]. In fact excellent results have been reported of practically all techniques. And although much emphasis should be given to the technique itself other factors related to the surgeon or to the patient and his pancreas may play an even greater role in the outcome after pancreatoduodenectomy. Patient-related factors as high age, and preoperative jaundice have shown a deleterious effect on anastomosic healing. Since morbidity and mortality in elderly patients because of anastomotic breakdown is very high[5] and the prognosis of pancreatic cancer is poor, a palliative (non-operative) procedure should be considered instead of pancreatoduodenectomy, for this group[10]. It has been suggested that the high incidence of anastomotic breakdown in jaundiced patients can be lowered by preoperative internal biliary drainage. And whereas percutaneous transhepatic external biliary drainage did not beneficially influence the outcome after surgery, partly because of the complications of the technique itself[11], endoscopic internal drainage has definitely earned its place in the preoperative treatment of jaundiced patients.

This procedure has also been advocated by Trede, who changed his opinion about performing a pancreatoduodenectomy in severely jaundiced patients[4]. The condition of the pancreas itself influences the success of pancreato-jejunostomy. The rate of leakage was 22% in patients with a soft, friable, normal pancreatic remnant and 6% in patients with a firm fibrotic pancreas[5]. A large diameter pancreatic duct has also a beneficial effect on the outcome of a pancreatic anastomosis[12]. It is obvious that patients undergoing a pancreatoduodenectomy for chronic pancreatitis do much better than patients with pancreatic cancer, since the former patients are younger, usually non-jaundiced, and have a firm pancreas with a dilated duct. Reports of the success of pancreato-enterostomies, considering both patients with malignant and benign disease should be read with the above mentioned in mind.

To circumvent the problems of the pancreatic-enteric anastomic, excision, ligation, stapling, duct occlusion or open drainage of the pancreatic remnant can be considered[8,13,14,15]. Total pancreatectomy has been advocated for the same reason and also because multicentricity of pancreatic cancer has been reported in up to 36%[16]. The hospital mortality of total pancreatectomy was rather high at first, although a decrease has been reported by Brooks[14]. This reported decrease together with the long-term survival of some patients with unsuspected foci of pancreatic cancer in the resected pancreatic tail make total pancreatectomy or, even more, the subtotal modification as described by Ahren *et al.*[1] worthwhile considering. Most authors, however, do not report an increased survival after total pancreatectomy[17] and the real drawback of this operation is the surgical diabetes mellitus, that leads to serious complications and even death by uncontrollable hypoglycemia[7,18,19]. Ligation, stapling, duct occlusion and open drainage are attractive, simple possibilities with acceptable mortality but a high morbidity according to the literature[8,12,13]. In the present publication under discussion subtotal pancreatectomy and stapling of the pancreatic remnant leads to a low complication rate. However, it can be argued that the delayed gastric emptying leading to prolonged hospitalisation in this series was a result of pancreatitis with infiltration as was actually demonstrated during relaparotomy in one patient. Non-drainage in the gastrointestinal tract will definitely result in exocrine pancreatic insufficiently, that is not always easy to treat and leads to malnutrition, especially in combination with gastric resection[2]. Furthermore, fibrosis as a result of some form of obstruction of the pancreatic duct will lead to endocrine pancreatic function as shown in experimental animals[20]. In 4 out of 7 long-term survivors this complication was seem after duct occlusion in one study whereas only 3 out of 39 patients had diabetes mellitus after pancreato-jejunostomy[15].

The use of stapling devices in pancreatectomy is a very attractive method since it is easy and gives a bloodless field. By removing a few staples the pancreatic duct can be opened and a pancreato-jejunostomy can be performed by the invaginating technique[20]. Two pancreatic fistulae were seen in 50 consecutive patients with a friable pancreas (Obertop, unpublished). As shown in the paper of Ahren *et al.* mortality of pancreatoduodenectomy has decreased over the last couple of years to a very acceptable level. Still the short-term and long-term morbidity is considered. Various modifications of the original procedure as reported by Whipple in 1985[21] have been proposed in order to improve the quality of life. The use of the pylorus preserving type of pancreatoduodenectomy seems one improvement[2]. Prevention of pancreatic insufficiency by use of pancreatoenteric anastomosis may well be another important feature in that respect, as long as this can be achieved with low risk by an experienced pancreatectomist.

### *References*

1. Ahren, B., Tranberg, K-G., Andren-Sanberg, A., Bengmark, S. Subtotal pancreatectomy for cancer: closure of the pancreatic remnant with staplers
2. Grace, P.A., Pitt, H.A., Tompkins, R.K., DenBesten, L., Longmire, W.P. (1986) Decreased morbidity and mortality after pancreatoduodenectomy. *Am. J. of Surg.* **151**: 141–149
3. Bloom, P., Steer, M.L. (1975). Pancreatoduodenectomy. Results when the operation is performed infrequently. *Arch. Surg.* **110**: 1455–1457
4. Trede, M., Schwall, G. (1988). The complications of pancreatectomy. *Ann. Surg.* **208**: 39–47
5. Lerut, J.P., Gianello, P.R., Otte, J.B., Kestens, P.J. (1984). Pancreaticoduodenal resection. Surgical experience and evaluation of risk factors in 103 patients. *Ann. Surg.* **199**: 432–437
6. Machado, M.C.C., Monteiro, D.A., Cunha, J.E., Bacchella, T. and others. (1976). A modified technique for the reconstruction of the alimentary tract after pancreatoduodenectomy. *Surg. Gynecol. Obstet.* **143**: 271–273
7. Funovics, J.M., Zoch, G., Wenzl, E. and Schulz, F. (1987). Progress in reconstruction after resection of the head of the pancreas. *Surg. Gynecol. Obstet.* **164**: 545–548
8. Icard, P., Dubois, F. (1988). Pancreaticogastrostomy following pancreatoduodenectomy. *Ann. Surg.* **207**: 253–256
9. Snellen, J.P., Obertop, H., Bruining, H.A., Eeftinck Schattenkerk, W.F., Eggink, W.F., Jeekel, J., van Houten H. (1985) The influence of preoperative jaundice, biliary drainage and age of postoperative morbidity and mortality after pancreatoduodenectomy and total pancreatectomy. *Neth. J. Surg.* **37**: 83–86
10. Pitt, H.A., Gomes, A.S., Lois, J.F., *et al.* (1985) Does preoperative percutaneous biliary drainage reduce operative risk or increase hospital cost? *Ann. Surg.* **201**: 545-553

11. Papachristou, D.N., Fortner, J.G. (1981). Management of the pancreatic remnant in pancreatoduodenectomy. *J. Surg. Oncol.* **18**: 1–7
12. Papachristou, D.N., D'Agostino, H., Fortner, J.G. (1980). Ligation of the pancreatic duct in pancreatectomy. *Br. J. Surg.* **67**: 260–262
13. Brooks, J.R., Books, D.C., Levine, J.D. (1989). Total pancreatectomy for ductal cell carcinoma of the pancreas. *Ann. Surg.* **209**: 405–410
14 Rovati, V., Brigante, G., Bevilacqua, M., Milanesi, A. (1984). Pancreatoduodenectomy with neoprene occlusion of main pancreatic duct. *The Lancet,* 1241–1242
15. Tryka, A.F., Brooks, J.R. (1979). Histopathology in the evaluation of total pancreatectomy for ductal carcinoma. *Ann. Surg.* **190**: 373–381
16. Longmire, W.P. (1984). Cancer of the pancreas: palliative operation, Whipple procedure, or total pancreatectomy. *World J. Surg.* **8**: 872–879
17. van Heerden, J.A., ReMine, W.H., Weiland, L.H., McIlrath, D.C., Ilstrup, D.M. (1981). Total pancreatectomy for ductal adenocarcinoma of the pancreas. *Am. J. Surg.* **142**: 308–311
18. Obertop,H., Bruining, H.A. Eeftinck Schattenkerk, M., Eggink, W.F., Jeekal, J. and van Houten, H. (1982). Operative approach to cancer of the head of the pancreas and the periampullary region. *Brit. J. Surg.* **69**: 573–576
19. Gooszen, H.G., Bosman, F.T., van Schilfgaarde, R. (1984). The effect of duct obliteration on the histology and endocrine function of the canine pancreas. *Transplantation* **38**: 13–17
20. Obertop, H., van Houten, H. (1984). A new technique for pancreatojejunostomy. *S.G.O.* **159**: 88–90
21. Whipple, A.D., Barclay Parsons, W., Mullins, C.R. (1935). Treatment of carcinoma of the ampulla of Vater. *Ann. Surg.* **102**: 763–779

Professor du H. Obertop
Department of Surgery
University Hospital, Utrecht, Netherlands

